# Proposal of Modified Lung-RADS in Assessing Pulmonary Nodules of Patients with Previous Malignancies: A Primary Study

**DOI:** 10.3390/diagnostics13132210

**Published:** 2023-06-29

**Authors:** Feipeng Song, Binjie Fu, Mengxi Liu, Xiangling Liu, Sizhu Liu, Fajin Lv

**Affiliations:** Department of Radiology, The First Affiliated Hospital of Chongqing Medical University, 1 YouYi Road, Chongqing 400010, China; 15834055634@163.com (F.S.); binjie_fu@163.com (B.F.); liumengxi_10@163.com (M.L.); m13996783778@163.com (X.L.); liusizhu98@163.com (S.L.)

**Keywords:** Lung-RADS, pulmonary nodules, computed tomography, X-ray, previous malignancy, modification

## Abstract

Background: In addition to the diameters of pulmonary nodules, the number and morphology of blood vessels in pure ground-glass nodules (pGGNs) were closely related to the occurrence of lung cancer. Moreover, the benign and malignant signs of nodules were also valuable for the identification of nodules. Based on these two points, we tried to revise Lung-RADS 2022 and proposed our Modified Lung-RADS. The aim of the study was to verify the diagnostic performance of Modified Lung-RADS for pulmonary solid nodules (SNs) and pure ground-glass nodules (pGGNs) in patients with previous malignancies. Methods: The chest CT and clinical data of patients with prior cancer who underwent pulmonary nodulectomies from 1 January 2018 to 30 November 2021 were enrolled according to inclusion and exclusion criteria. A total of 240 patients with 293 pulmonary nodules were included in this study. In contrast with the original version, the risk classification of pGGNs based on the GGN–vascular relationships (GVRs), and the SNs without burrs and with benign signs, could be downgraded to category 2. The sensitivity, specificity, and agreement rate of the original Lung-RADS 2022 and Modified Lung-RADS for pGGNs and SNs were calculated and compared. Results: Compared with the original version, the sensitivity and agreement rate of the Modified version for pGGNs increased from 0 and 23.33% to 97.10% and 92.22%, respectively, while the specificity decreased from 100% to 76.19%. As regards SNs, the specificity and agreement rate of the Modified version increased from 44.44% to 75.00% (*p* < 0.05) and 88.67% to 94.09% (*p* = 0.052), respectively, while the sensitivity was unchanged (98.20%). Conclusions: In general, the diagnostic efficiency of Modified Lung-RADS was superior to that of the original version, and Modified Lung-RADS could be a preliminary attempt to improve Lung-RADS 2022.

## 1. Introduction

Cancer has been the leading case of deaths in China and developed countries [1,2], and has been the second cause of deaths in the United States [3]. On the premise that the overall 5-year survival rate of cancer is generally rising, along with the development of cancer screening technology and the continuous progression of cancer treatment, the numbers of the special group with previous malignancies are also increasing year by year [4,5]. Relevant research data shows that the probability of developing a second primary lung cancer (SPLC) is also increasing in this group [5,6].

As for lung cancer, it is projected to become the leading cause of cancer death in China and the United States by 2022 [7], while the prognosis and mortality of patients with previous histories of cancer suffering from SPLCs are not different from those of patients without prior malignancies [8,9]. Low-dose CT (LDCT), as the main screening method for lung cancer, can effectively reduce the mortality of lung cancer [10,11,12]. However, not all cancer survivors are eligible for lung cancer screening, even though a history of malignancy is a risk factor, and such patients often get regular chest CT examinations after their initial cancer diagnoses. Therefore, the question of how to assess risk and conduct the follow-up management of pulmonary nodules when these people undergo chest screening or oncologic surveillance has always been a difficult issue faced by clinicians and radiologists.

Lung-RADS, created by the American College of Radiology (ACR) in 2014 and updated in November 2022 [13], served as a standardized nodule follow-up management paradigm for lung cancer LDCT screening reports and played an important role in the risk assessment and management of pulmonary nodules in clinical practice. In addition, Lung-RADS, as applied by radiologists in clinical practice, achieved excellent performance on follow-up screening examinations [14]. However, it also had certain defects [15], especially for sub-solid nodules. Lung-RADS could underestimate the risk of malignancy in such nodules and had poor prediction ability compared with the Brock risk calculator and Vancouver risk calculator [16,17,18]. In addition, the imaging signs of nodules were ignored by Lung-RADS when focusing on the classification by diameter or volume, and the description of additional imaging features in the category 4X was not specific, resulting in many inconsistencies in the determination of the category 4X and a certain false positive rate in clinical application [19,20]. Furthermore, whether these deficiencies also existed in the assessment of pulmonary nodules (PNs) in patients with histories of previous cancer was still unclear.

Previous studies [21,22] had confirmed that the amount and the morphology of GGN vessels were closely related to the occurrence of lung cancer. Gao et al. [23] divided the spatial relationship between GGNs and blood supply vessels (GVR) into four types and found that it was valuable to differentiate GGNs. Additionally, Qing et al. [24] demonstrated that their Complementary Lung-RADS 1.1, based on GVRs for risk stratification for GGNs in lung cancer screening, was superior to Lung-RADS 1.1. Therefore, we presented our modification scheme of Lung-RADS 2022 for GGNs based on GVRs. As we knew, pulmonary nodule size was an independent predictor of malignancy [25]. However, the imaging findings of nodules could not be ignored either. There are many malignant signs of lung cancer, but not all nodules with malignant signs indicate lung cancer. Chen et al. [26] found that there were no significant differences in vacuole sign, vessel convergence, and pleural depression sign between benign and malignant pulmonary nodules. However, burr sign and calcification components were diverse in the benign and malignant groups. For this reason, we attempted to downgrade the malignant risk of solid nodules according to the presence of benign signs.

Based on these two viewpoints, we initially proposed Modified Lung-RADS and compared it with Lung-RADS 2022 in terms of diagnostic efficiency for PNs, as explained in the following sections.

## 2. Patients and Methods

### 2.1. Patients

The chest CT images of patients with previous cancers who had undergone surgical resections of PNs in our hospital from 1 January 2018 to 30 November 2021 were collected and retrospectively analyzed. ‘Previous malignancy’ was defined as a cancer that had been diagnosed before the pulmonary nodules were found in a patient’s lungs.

Inclusion criteria: 

(1) patients ≥ 18 years old; 

(2) a definite history of previous malignancy; 

(3) size of SN: ≤30 mm; the sizes of pGGNs were not limited; 

(4) the data was complete and the slice thickness was less than 1.5 mm; 

(5) the final pathological results were definite; the length of the cancer history was not limited; 

(6) the previous malignancies originated from parenchymal organs and the haematological system.

**Figure 1:**
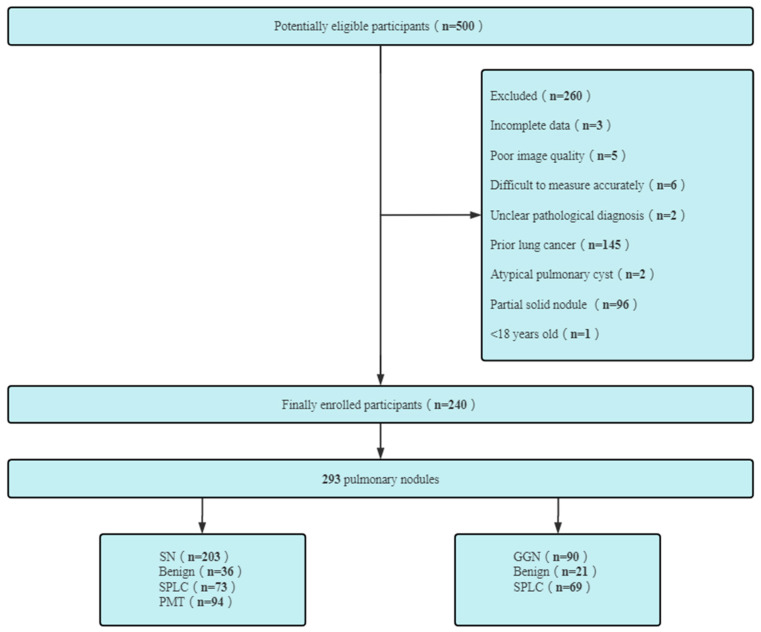
SN—solid nodule; GGN—ground glass nodule; SPLC—second primary lung cancer; PMT—pulmonary metastatic tumor.

Exclusion criteria: 

(1) partial solid nodules; atypical pulmonary cyst;

(2) the quality of the image was poor, and there were respiratory motion artifacts that interfered with the image diagnosis; 

(3) nodules were so close to the hilum or were closely related to hilum structure so that the sizes of nodules could not be accurately measured; 

(4) the patient suffered from obstructive pneumonia, atelectasis, pneumothorax, or massive pleural effusion; 

(5) time interval of preoperative CT > 3 months; 

(6) patients with prior lung cancer; patients undergoing chemo or immunotherapy. Figure 1 presents the exclusion criteria and the patient recruitment process.

The time interval of preoperative CT was defined as the length between the time of the last preoperative CT examination and the date of surgery.

### 2.2. CT Protocol

The non-contrast chest CT scans were performed using the SOMATOM Definition Flash (Siemens Healthineers, Erlangen, Germany), SOMATOM Force (Siemens Healthineers, Erlangen, Germany), and Discovery CT750 HD (GE Healthcare, Milwaukee, WI, USA) CT scanners. All the patients were asked to place their hands over their heads in supine positions, take deep breaths, and hold their breaths. The scan range was from the tip of the lung to the level of the costophrenic angle. The protocol parameters were as follows: tube voltage—100–120 kV; tube current—30–50 mA; slice thickness—5 mm; reconstruction slice thickness—1 mm; matrix—512 × 512; rotation speed—0.5 or 0.6 s/r; pitch—1 or 0.984.

### 2.3. Image Analysis

#### 2.3.1. Observation and Measurement of Nodules

All images of PNs were read by two radiologists on the picture archiving and communication system in a blind manner. CT image window width (WW) and window level (WL) were set thus: lung window-WW—1500 Hu, WL—600 Hu; mediastinal window-WW—300 Hu, WL—60 Hu. The signs and sizes of nodules were observed and measured on a thin image, and the diameters (i.e., the averages of the long and short diameters) of PNs were measured at the lung windows, usually at the transverse slices, unless the longest diameter of the nodules were in the coronal or sagittal position [27].

Imaging classification of PNs: An SN is defined as a lesion whose density was higher than that of the blood vessels and could be seen in the mediastinal window. ‘pGGN’ refers to the low-density nodules that cannot cover the passing vessels in the lung window.

Findings on SNs: Benign signs include fibrous cords around the lesion, a blurred boundary, patchy exudation shadows around the lesion, a calcification of the nodules, and surrounding satellite lesions. Lobations, spiculations, pleural indentations, vascular convergences, and vacuoles are malignant signs.

**Figure 2:**
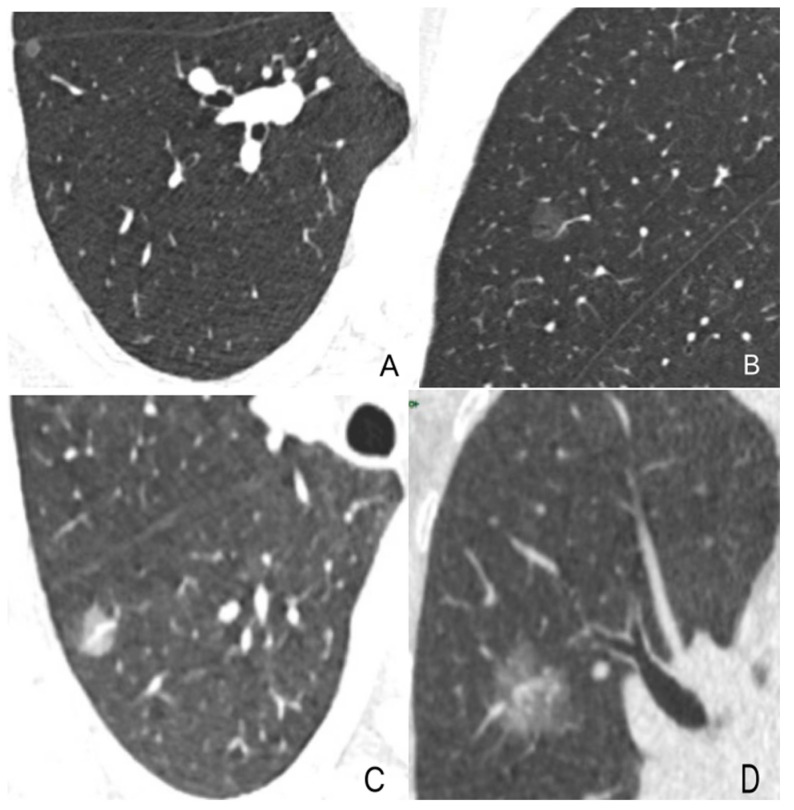
Categories of GVR: Type I GVR (**A**), vessels passing by lesions without any detectable supplying branches to the lesions; Type II GVR (**B**), vessels passing through the pGGNs without obvious morphological changes in traveling path or size; Type III GVR (**C**), vessels within nodules appearing tortuous or rigid without an increase in amount; Type IV GVR (**D**), more complicated vasculature within nodules than the 3 types mentioned above, such as in the coexistence of irregular vascular dilation and vascular convergence from multiple supplying vessels.

GGNs and vascular relationships (GVR) [23,24]: There are four types of GGNs according to their different vascular features, as shown in Figure 2.

#### 2.3.2. Category of Pulmonary Nodules

A negative screen was defined as comprising categories 1 and 2, and a positive screen was defined as comprising categories 3 and 4. Meanwhile, the pathological diagnosis of a malignant tumor was defined as positive, and that of a benign lesion was defined as negative.

First of all, SNs and GGNs were classified by Lung-RADS 2022 [13]. Then, based on the presence of benign signs of solid nodules and the different kinds of GVRs [23,24], the original Lung-RADS 2022 were improved to obtain Modified Lung-RADS. GGNs and SNs were reclassified by Modified Lung-RADS, as shown in Table 1 with the details.

### 2.4. Statisticalanalysis

The sensitivity, specificity, and agreement rate (AR) of Lung-RADS 2022 in the diagnosis of PNs were calculated according to the pathological diagnosis. All the nodules were reclassified according to Modified Lung-RADS, and the sensitivity, specificity, and AR were calculated and compared with the original Lung-RADS 2022 by using chi-square test with SPSS (version 22.0). The consistency between two radiologists was conducted by a Kappa test. *p* < 0.05 was statistically significant.

## 3. Results

### 3.1. General Data Statistics of Patients

A total of 240 patients with 293 pulmonary nodules were included in this study. There were 83 males (62.0 ± 11.7 years, range 22–81 years) and 157 females (56.1 ± 10.0 years, range 32–84 years). All PNs were composed of 203 solid nodules (benign 36, malignant 167) and 90 GGNs (benign 21, malignant 69), as shown in Figure 1.

The patients with previous cancers included 46 cases of the head and neck, 55 cases of the breast, 72 cases of the digestive system, 12 of the urinary system, 34 of the genital system, 3 of the skeletal and muscular system, and 6 cases of others. In addition, there were 12 patients with more than 2 kinds of prior malignancy.

A total of 12 patients (5.0%) had never been smokers and 235 cases (95.0%) had smoking histories (mean pack years: 38.9 (range 0–125)). A total of 29 patients had pack year histories of <20 years. A total of 3 individuals claimed to have quit smoking (cease-smoking time range: 2 months–10 years).

**Figure 3:**
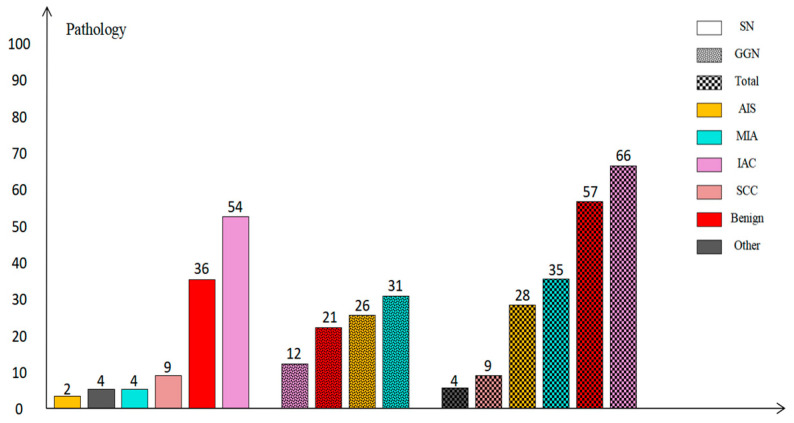
Summary of pathological diagnosis of pulmonary nodules. SN—solid nodule; GGN—ground-glass nodule; IAC—invasive adenocarcinoma; MIA—micro-invasive adenocarcinoma; SCC—squamous cell carcinoma; AAH—atypical adenomatous hyperplasia; AIS—Adenocarcinoma in situ.

**Figure 4:**
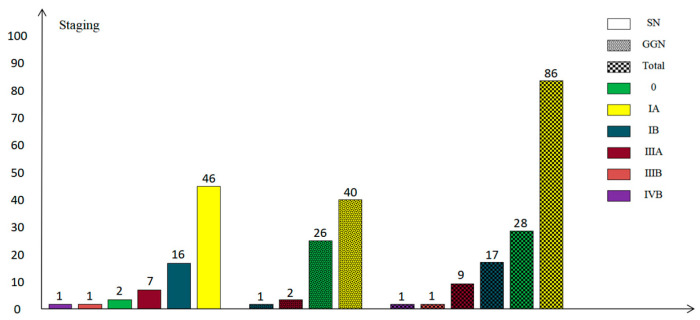
Summary of clinical staging of pulmonary nodules. SN—solid nodule; GGN—ground-glass nodule.

The most common histological types of SPLCs in the present study were invasive adenocarcinoma (46.5%, 66/142), micro-invasive adenocarcinoma (24.6%, 35/142), and adenocarcinoma in situ (19.7%, 28/142), and the most common clinical stage was IA (60.6%, 86/142) (see Figure 3 and Figure 4).

#### 3.1.1. Consistency between Observers

All pulmonary nodules were evaluated according to the original version and the Modified version of Lung-RADS, and the Kappa values of the two radiologists were 0.81 and 0.87, respectively, with *p* < 0.001.

#### 3.1.2. Comparison of Diagnostic Accuracy between Original and Modified Versions for pGGN

Compared with the original version, the sensitivity and AR of Modified Lung-RADS for pGGNs increased from 0 and 23.33% to 97.10% and 92.22%, respectively, while the specificity decreased from 100% to 76.19%, as shown in Table 2.

#### 3.1.3. Difference of Diagnostic Performance of SNs between Original and Modified Versions

From Table 3, it is not difficult to find that compared with the original version, the specificity and AR of Modified Lung-RADS for SNs increased from 44.44% to 75.00% (*p* < 0.05) and from 88.67% to 94.09% (*p* = 0.052), respectively, while the Se was stable (98.20% vs. 98.20%).

## 4. Discussion

The chances of developing a second primary lung cancer are different among different previous cancers [28]. In our data, the cases of second primary lung cancer occurred the most in previous cancers of the digestive system, followed by the incidences of breast and head and neck cancers, a finding which was close to that in the previous literature [28,29]. Moreover, Bertoglio et al. [30] showed that the presence of previous cancer did not have a strong influence on the overall survival of second primary lung cancer compared with patients without prior malignancies. This suggested that it was also necessary to screen lung CT for such patients with histories of malignant tumors in clinical practice. Although NLST (National Lung Screening Trial) has a clear definition of ‘individual at high risk’ for lung cancer screening, this is not always the case in clinical practice due to the preference of individuals or clinicians. In addition, different countries and regions have different definitions of high-risk groups of lung cancer. For example, in China, high-risk groups of lung cancer are defined as those who are at least 40 years old and have any risk factors of lung cancer [31], such as previous cancer histories. Moreover, it was reported that some high-risk patients not meeting the NLST inclusion criteria may benefit from lung cancer screening [32]. Halpenny et al. [33] conducted lung cancer screening for patients with previous histories of malignancy by using Lung-RADS, and their enrolled cases did not fully meet the criteria for high-risk groups provided by the NCCN (National Comprehensive Cancer Network). Therefore, the ages and smoking histories of enrolled individuals were not strictly required in the present research. In addition, since this was a preliminary exploratory study, the indicators we compared were objective items such as nodule diameter and Lung-RADS category, which would not affect the reliability of the results of this study.

Except for the case of a single prior cancer, it was worth noting that patients with multiple primary cancers also occupied a certain proportion, accounting for about 5.0% (12/240) of all patients in our research. Copur et al. [34] showed that overall reported frequencies of multiple primary cancers varied between 2.4% and 17%, and the incidence of our statistics was also within this range. The nodules enrolled in our study were not only solitary pulmonary nodules, but additionally, multiple pulmonary nodules in the same patient, which could be benign or signs of malignant disease at the same time, were included. Therefore, we recommended that each nodule should be evaluated separately, and this was also the suggestion of the Fleischner Society [35]. According to the final pathological diagnosis of all enrolled cases, 83.8% (201/240) of the patients suffered from malignant pulmonary nodules and 57.5% (138/240) of the patients suffered from only the second primary lung cancer, a finding that was much higher than that in the statistical results of O’Dwyer et al. [36]. The reason for this should be related to the fact that all cases included in our study were patients who underwent surgical resection and that PSNs were excluded.

According to our previous statistical results, the sensitivity, specificity, and agreement rate of Lung-RADS 2022 for partial solid nodules in patients with previous histories of cancer were all greater than 90% (not shown in this paper). This indicated that Lung-RADS 2022 can be used to evaluate pulmonary partial solid nodules in patients with histories of malignancy, and so, the PSNs were not further studied in our study. Moreover, the number of atypical pulmonary cyst was too small (only 2) to conduct separate statistical analysis, and so they were also excluded from the current study. However, our research objects were SNs and pGGNs.

The pulmonary pGGNs and PSNs all belonged to the category of sub-solid nodules. However, in contrast to PSNs, the assessment of pGGNs by Lung-RADS 2022 showed the worst diagnostic performance in patients with histories of cancer, with extremely low sensitivity and a high risk of false negative diagnosis. The data of our research showed that malignant lesions accounted for 76.7% (69/90) of all pGGNs, and the proportion of invasive adenocarcinoma was as high as 13.3% (12/90). This was consistent with the finding that Lung-RADS underestimated the malignant risk of pGGNs, which had been reported in related studies [17,18]. Therefore, Lung-RADS was also not suitable for the assessment of pGGNs in patients with prior malignancies.

In order to resolve this defect of Lung-RADS, we re-evaluated GGNs by using Modified Lung-RADS based on GVRs reported in the literature [23,24]. Compared with the original Lung-RADS 2022 published by ACR, it was found that the sensitivity and agreement rate of Modified Lung-RADS for pGGNs were significantly improved, and the sensitivity increased from 0 to 97.1% and the agreement rate increased from 23.3% to 92.2%. This was close to what had been reported in the literature [24], which makes up for the shortcomings of the original Lung-RADS in the diagnosis of pGGNs with a high rate of missed diagnosis and poor diagnostic efficiency. Additionally, the specificity was 76.2%, which was significantly higher than that reported in the previous study [24]. It was speculated that the reason may be related to the relatively small sample of pGGNs included in their study—only 49 nodules. Nor did it exclude that the population category in the literature was inconsistent with that in our study, and that the cases included in our study all underwent surgical resection, which may lead to a certain selection bias. Although the sensitivity and agreement rate of the Modified version were satisfactory, combined with clinical practice, it was found that the specificity in our study was less than 80% and the false positive rate was more than 20%. In other words, about one-fifth of patients with benign pGGNs will undergo surgical resection, so the question of how to further improve specificity still needs to be explored in the future.

As for solid nodules in the patients with previous malignancies, it was found that there was a high diagnostic accuracy of Lung-RADS 2022 in our study, with a sensitivity of 94.81% and an agreement rate of 86.74%, which was close to the data based on the general population [16]. However, further analysis revealed that Lung-RADS 2022 had a high false positive rate for SNs in our research. The statistical results of our study showed that the false positive rate of Lung-RADS 2022 for solid nodules was as high as 55.6%, which was much higher than the results of some studies based on the general population [16,37]. When we further analyzed the causes, it was found that such false positive solid nodules tended to have large sizes, with an average size of 10.6 mm, a minimum size of 6.0 mm, and a maximum size of 22.8 mm. Patients with previous histories of cancer often had a certain psychological burden and great fear or anxiety about malignant tumors. Therefore, after pulmonary nodules were found, they often chose non-standard short-term review or sought active surgical treatment. In addition, a study had shown that the probability of absorption and dissipation in chest CT follow-up after the anti-inflammatory treatment of solid nodules was much lower than that for partial solid nodules and pure ground-glass nodules, which was only 22% [38]. As thoracic surgeons, they may have also been inclined to perform surgical resection in such patients when there was no obvious absorption in chest CT follow-ups after anti-inflammatory therapy. All the factors mentioned above may jointly lead to an increase in false positive rates.

**Figure 5:**
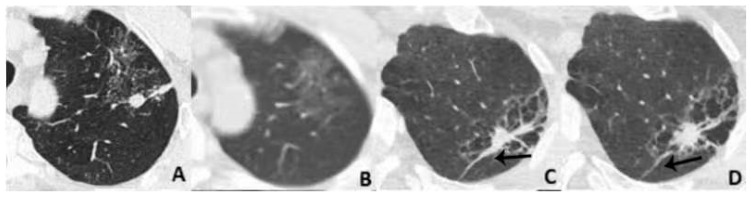
SNs reclassified by Modified Lung-RADS 2022. (**A**,**B**) Male, 77y, previous sigmoid colon cancer; a new SN of about 7.4 mm was detected in the upper lobe of the left lung (**A**), with a clear nodule boundary and cord around it. Patchy exudative lesions were significantly absorbed compared to a CT image from 6 months ago (**B**). The scores of Lung-RADS 2022 and Modified Lung-RADS 2022 were 4A and 2, respectively. Finally, the pathological diagnosis was tuberculoma. (**C**,**D**), male, 77y, prior thyroid carcinoma; a lesion with a size of 16.0 mm in the left upper lobe was found 11 days ago. Spiculation, lobulation, and pleural indentation around the nodule could be seen, and additionally, there were cord shadows (arrow) on the largest (**D**) and upper layers (**C**) of the nodule. The categories of the original version and Modified version were both 4X. Invasive adenocarcinoma was confirmed by pathology.

In order to reduce the false positive rate as much as possible, we also analyzed the imaging manifestations of false positive solid nodules and found that about half of the solid nodules included in our study had different benign signs such as fibrous cords, benign calcification, a blurred boundary, patchy exudation shadows, satellite lesions, and so on. Among the true positive solid nodules, there was only one solid nodule with a benign sign, and none were found in the other malignant SNs. This was the source of our idea to improve Lung-RADS 2022 according to the existence of benign signs. According to the ACCP (American College of Chest Physicians) and Fleischner Society guidelines [35,39], nodules with marginal spiculation indicate a risk factor for lung cancer. Therefore, we tried to downgrade SNs of Category ≥ 3 without spiculation signs and with more than 1 benign sign to Category 2 by using Modified Lung-RADS (Figure 5A,B). If a solid nodule had a spiculation sign, its Lung-RADS category remained unchanged regardless of whether there were benign signs (Figure 5C,D). According to this modified scheme, only positive SNs classified by the original Lung-RADS 2022 were reclassified. In our study, it was found that the specificity were significantly improved, and none of the malignant nodules were incorrectly downgraded to negative nodules. In other words, our Modified version could significantly reduce the probability of unnecessary surgical resection in patients with false positive SNs, and did not decrease the original’s high sensitivity and agreement rate.

There were also some limitations in our study. Firstly, our research was a single-center retrospective study, and Modified Lung-RADS, proposed by us, still needs further multi-center and even prospective verification in the future. Secondly, the individuals included in our study were all patients who had undergone surgical resection, which may indicate a certain selection bias. Thirdly, although the number of cases in our study was large, the number of solid nodules determined to be positive according to Lung-RADS was relatively small. The results may be biased to some extent, and the sample size needs to be further expanded in the future.

## 5. Conclusions

In conclusion, compared with the original Lung-RADS 2022, diagnostic efficiency was improved in Modified Lung-RADS. The improved version could be used as a preliminary attempt to reform Lung-RADS and also needs to be further verified in clinical practice.

## Figures and Tables

**Table 1:** The original and Modified Lung-RADS for SNs and GGNs.

Category	Solid Nodule (SN)		Pure Ground Glass Nodule (pGGN)	
	OV	MV	OV	MV
2	<6 mm at baseline OR new < 4 mm Juxtapleural nodule: <10 mm mean diameter at baseline or new AND Solid; smooth margins; oval, lentiform, or triangular shape	<6 mm at baseline OR new < 4 mm; Category 3 or 4 nodules without spiculation and with additional features (≥1 sign) that indicate the suspicion of benign disease; Category 3 nodules unchanged for ≥6 months Juxtapleural nodule: <10 mm mean diameter at baseline or new AND solid; smooth margins; oval, lentiform, or triangular shape	<30 mm at baseline, new, or growing OR ≥30 mm stable or slow-growing	<30 mm and type I GVR; Category 3 or 4 nodules stable ≥ 5 years; Category 3 or 4 nodules decreased in size and there was an absence of solid components OR this was resolved on a follow-up
3	≥6 to <8 mm at baseline; new 4 mm to <6 mm; Category 4A nodule that is stable or decreased in size at 3-month follow-up CT (excluding airway nodules)	≥6 to <8 mm at baseline; new 4 mm to <6 mm Category 4A nodules unchanged for ≥3 months	≥30 mm at baseline or new	≥30 mm and type I GVR; any size with type II GVR
4A	≥8 to <15 mm at baseline OR Growing < 8 mm OR New 6 to <8 mm	≥8 to <15 mm at baseline; growing < 8 mm; new 6 to <8 mm	-	any size with type III GVR
4B	≥15 mm at baseline OR New or growing ≥ 8 mm	≥15 mm at baseline; new or growing, and ≥8 mm	-	any size with type IV GVR
4X	Category 3 or 4 nodules with additional features or imaging findings that increase suspicion for lung cancer	Category 3 or 4 nodules with additional features or imaging findings that increase the suspicion of malignancy and lack benign signs; Category 3 or 4 nodules with spiculation sign with or without benign signs	Category 3 or 4 nodules with additional features or imaging findings that increase suspicion for lung cancer	Category 3 or 4 nodules with additional features or imaging findings that increases the suspicion of malignancy

OV—original version; MV—Modified Version; GVR—GGN–vascular relationship.

**Table 2:** Comparison of diagnostic accuracy of pGGNs between two versions of Lung-RADS.

	OV	MV	χ^2^	*p*
TP	0	67	-	-
FP	0	5	-	-
TN	21	16	-	-
FN	69	2	-	-
Sensitivity (%)	0	97.10	-	-
Specificity (%)	100	76.19	-	-
AR (%)	23.33	92.22	87.54	<0.001 ^#^

Abbreviations: OV—original version; MV—Modified version; TP—true positive; FP—false positive; TN—true negative; FN—false negative; AR—agreement rate; # Pearson Chi-Square test.

**Table 3:** Comparison of diagnostic accuracy of SNs between two versions of Lung-RADS.

	OV	MV	χ^2^	*p*
TP	164	164	-	-
FP	20	9	-	-
TN	16	27	-	-
FN	3	3	-	-
Sensitivity (%)	98.20	98.20	-	-
Specificity (%)	44.44	75.00	-	0.002 *
AR (%)	88.67	94.09	3.78	0.052 ^#^

Abbreviations: OV—original version; MV—Modified version, * Fisher’s exact probabilities; # Pearson Chi-Square test; TP—true positive; FP—false positive; TN—true negative; FN—false negative; AR—agreement rate.

## Data Availability

The datasets used and/or analysed during the current study are available on request from the corresponding author.
